# Fano Resonant Sensing in MIM Waveguide Structures Based on Multiple Circular Split-Ring Resonant Cavities

**DOI:** 10.3390/mi16020183

**Published:** 2025-02-03

**Authors:** Wenjing Wang, Shaoze Zhang, Huiliang Cao

**Affiliations:** 1 School of Microelectronics, University of Science and Technology of China, Hefei 230022, China; w520480@126.com; 2 East China Institute of Photo-Electron IC, Bengbu 233000, China; 3 School of Instrument and Electronics, North University of China, Taiyuan 030051, China

**Keywords:** fano resonance, plasmon refractive index nanosensor, finite element method (FEM), sensitivity, figure of merite (FOM)

## Abstract

In this work, a non-through metal–insulator–metal (MIM) waveguide capable of exciting three Fano resonances was designed and numerically studied using the finite element method. Fano resonances are achieved through the interaction between the modes of multiple circular split-ring resonator cavities and the waveguide. The effect of coupling between different resonators on the Fano resonance peaks is investigated. Independent tuning of the Fano resonance wavelength and transmission rate is accomplished by modifying the structural rotation angle and geometric parameters. After optimizing these parameters, the structure achieves an optimal refractive index sensitivity of 946.88 nm/RIU and a figure of merit of 99.17. The proposed structure holds potential for guiding the design of nanosensors.

## 1. Introduction

The Fano resonance phenomenon has a wide range of applications in optics, particularly in the fields of sensing and detection, such as in lasing [1], biological [2] and chemical sensing [3], switching [4], enhancing the slow light effect [5], surface-enhanced Raman scattering (SERS) [6], and enhanced infrared light absorption [7]. The unique characteristics of Fano resonance confer superior performance in these areas, including high sensitivity [8], rapid response [9], and the ability to achieve highly tunable responses [10]. Notably, when Fano resonance is combined with other optical effects, it can significantly enhance signal contrast [11] and resolution [12], thereby greatly improving the detection capability of sensors. Among the various approaches for realizing Fano resonance, the metal–insulator–metal (MIM) waveguide structure [13,14] has garnered significant attention due to its high integration at the micro/nanoscale and excellent optical properties. MIM waveguides exhibit strong local electromagnetic field enhancement, which aids in the formation of Fano resonance [15], and their resonant characteristics can be tuned by altering the geometry of dielectric layers [16] and material properties of the metal [17]. Due to their excellent light field localization and low losses, MIM waveguides are particularly suitable for applications requiring high sensitivity and precise control.

In particular, Fano resonance phenomena realized through MIM structures can be applied in sensing applications within the near-infrared region [18]. Existing studies can be broadly categorized into the following areas: first, sensor designs based on MIM waveguides have focused on the impact of waveguide geometry [19], metal material selection [20], and dielectric layer thickness [21] on the characteristics of Fano resonance. By optimizing these parameters, researchers have been able to achieve more precise wavelength tuning, thereby enhancing the sensor’s response sensitivity in the NIR region [22]. Second, some studies have explored the interaction between Fano resonance and biomolecules, developing high-sensitivity sensors for biomolecule detection based on the spectral characteristics of Fano resonance [23]. Additionally, research has been conducted on the application of MIM structures on integrated optical platforms, where miniaturization and integration techniques have enabled the development of efficient, low-cost sensor platforms suitable for large-scale sensor networks and field detection [24].

The advantages of MIM waveguides are not only reflected in their local light enhancement effects but also in their design flexibility. Variations in metal materials, dielectric layer thickness, and metal layer geometry can significantly influence the transmission properties of the waveguide [25], providing a wide range of tuning options for the design of optical sensors. Researchers have proposed different MIM waveguide structures to achieve Fano resonance and enhance sensing performance. In 2019, Qiao et al. proposed a metal–insulator–metal (MIM) waveguide structure consisting of an M-type cavity and a baffle with a sensitivity of up to 780 nm/RIU, and the FOM of the structure was $1.5\times 10^{6}$ [26]. In 2020, Zhu et al. proposed a unilaterally coupled metal–insulator–metal (MIM) structure using surface plasma and a U-shaped cavity with a sensitivity of up to 825 nm/RIU; although its transmittance could reach 0.9, the FOM of the structure was 21.54 and 19.92 [27]. In 2020, Liu et al. proposed a metal–insulator–metal (MIM) waveguide structure consisting of a side-coupled rectangular cavity (SCRC), a rightward opening semi-ring cavity (ROSRC), and a bus waveguide. The maximum sensitivity of refractive index sensing using this structure was up to 1550.38 nm/RIU, and the FOM of the structure was 7358, but its transmittance was only about 0.4 maximum [28]. In 2021, Xiao et al. proposed an MIM waveguide structure composed of inverted U-shaped and triangular groove cavities with a sensitivity of up to 840 nm/RIU and an FOM of the structure of $3.9\times 10^{5}$, but its transmittance was only about 0.3 maximum [29]. The structure presented in this paper has a sensitivity of 946.88 nm/RIU, an FOM of 99.17, and a transmittance of 0.8; although the parameters of some of the definitions of the above structures have higher values than those of the structure in this study, the three parameters of the structure presented in this study have higher values overall. In this study, a structure consisting of an MIM waveguide and multiple circular split-rings is designed and the transmission spectrograms and magnetic field distribution of the structure are simulated using the finite element method (FEM). However, our main contribution is in the innovation of the structure. Since two structures, a circular split-ring [30] and coupled multiple circular rings [22], have previously been studied, and in [16], only the properties of square split-ring structures are discussed, this thesis focuses on comparing the transmission spectra maps of different resonant cavities coupled with a circular split-ring and varying the key parameter, the rotation angle of the structure, and reveals the mechanism of the structure. The sensitivity and FOM of the structure are explored, and finally the effect of changing the structural parameters on the transmission spectrograms is explored, which will be helpful for the understanding and application of the sensor in the future.

## 2. Structural Modelling and Analytical Methods

**Figure 1:**
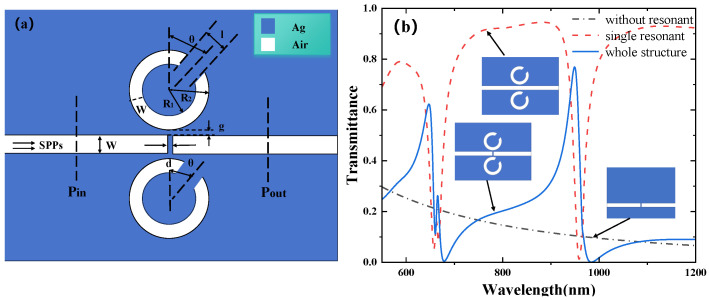
(**a**) Two-dimensional schematic of an MIM waveguide with the double CSRRCs; (**b**) finite element method (FEM) calculations of transmission spectra of the overall structure, no resonant cavity, and a single resonant cavity.

The schematic diagram of the designed waveguide structure is shown in Figure 1a. Simulations of this structure were performed using COMSOL software. The structure consists of multiple circular split-ring resonant cavities and an MIM waveguide with a baffle. The width of both the MIM waveguide and the circular split-ring resonance cavity (CSRRC) is fixed at W=50 nm. The CSRRC is formed by splitting a complete ring, with the inner and outer radius of the CSRRC being $R_{1}=90$ nm and $R_{2}=140$ nm, respectively. The structural parameters of the CSRRCs are identical, with the upper CSRRC defined as Resonator 1 and the lower CSRRC as Resonator 2. The coupling gap between the CSRRCs and the waveguide is set to g=10 nm, and the baffle width is d=10 nm. While a 3D model requires substantial hardware and meshing resources, it was found that the magnetic field distribution at each cross-section remains consistent regardless of whether the model is constructed in 2D or 3D. This indicates that the magnetic field distribution is not affected by the dimensionality of the model. Therefore, to optimize computational efficiency, a 2D model was used for the simulations. In Figure 1, the blue region represents the metal (silver), while the white region represents the air filling. Silver was chosen as the material for the filling layer due to its relatively low relative permittivity and strong electromagnetic confinement capabilities. Additionally, silver’s low power loss and strong ability to constrain the electromagnetic field make it a suitable choice.

During simulations using the finite element method, the relative dielectric constant of metallic silver can be described by the Drude model [31]:

(1) $$\varepsilon_{Ag}=\varepsilon_{\infty }-\frac{\omega_{P}^{2}}{\omega^{2}-i\Gamma \omega }$$

In Equation (1), the dielectric constant of the infinite frequency is taken as $\varepsilon_{\infty }=3.7$, plasma oscillation frequency as $\omega_{P}=9.1$ eV, and damping coefficient as Γ=0.018 eV, while ω represents the angular frequencyofthe incident wave.

**Figure 2:**
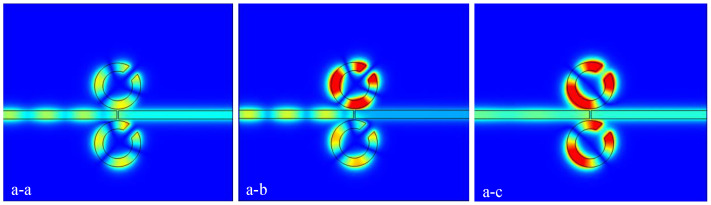
(**a-a**) Magnetic field distribution of the structure at λ=645 nm; (**a-b**) magnetic field distribution of the structure at λ=665 nm; (**a-c**) magnetic field distribution of the structure at λ=950 nm.

To analyze the optical response characteristics of the coupled structure, a geometric model was designed using COMSOL software. A fine mesh grid was employed to ensure convergence. Certain parameters, such as the relative permittivity of silver, the refractive index of air, and the top and bottom boundaries of the structure, were set using perfectly matched layers (PML). The transmission spectrum of the coupled system was obtained through simulation. Surface plasmon polaritons (SPPs) were excited by transverse magnetic (TM) [32] polarized light and propagated along the bus waveguide. The input and output powers were measured as Pin and Pout, respectively, and the transmission spectrum was calculated using the formula T=Pout/Pin. The structure was simulated using COMSOL, and the results are shown in Figure 1b. From the simulation results, it can be observed that in the case of the MIM waveguide with only a baffle, the transmission curve of the structure, represented by the black line in the figure, appears as a wide continuous state. In the case of only the CSRRCs, the transmission curve is represented by the red line, showing a narrow discrete state. Through the mutual coupling of the two modes, three asymmetric Fano resonance peaks were formed. The first Fano resonance peak occurs at a wavelength of 645 nm, with a transmission rate of approximately 61.42%; the second peak occurs at 665 nm, with a transmission rate of about 26.23%; and the third peak occurs at 950 nm, with a transmission rate of around 76.73%, as shown by the blue curve in Figure 1b. Figure 2 shows the magnetic field distributions for the first, second, and third Fano resonance peaks, with the energy being concentrated around the resonators.

## 3. Simulation and Analysis of Results

### 3.1. Analysis of the Rotation Direction of the CSRRC

**Figure 3:**
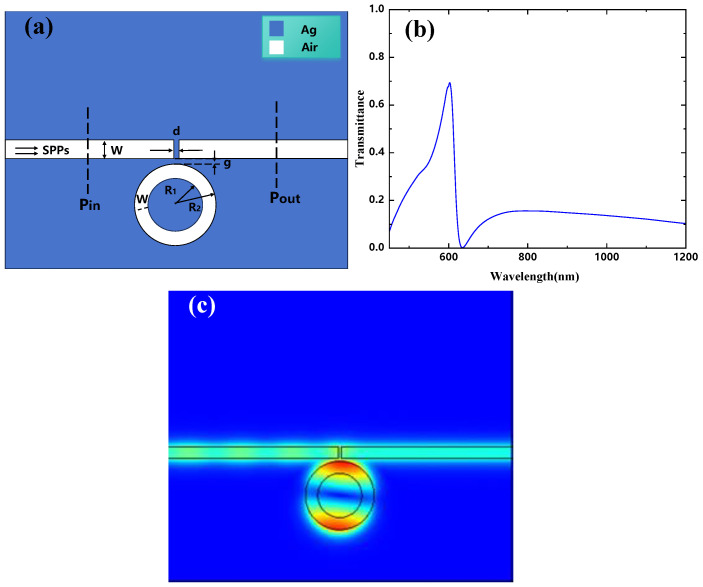
(**a**) Two-dimensional schematic of the MIM waveguide for Resonator 3; (**b**) finite element method (FEM) calculation of the transmission spectrum for Resonator 3; (**c**) magnetic field distribution on Resonator 3.

Before analyzing the structural dependence on the rotation direction of the CSRRC, the results for the complete ring positioned on the side of the waveguide are first examined [22]. The complete ring is defined as Resonator 3, with the structure shown in Figure 3a, where the parameters are $R_{1}=90$ nm, $R_{2}=140$ nm, W=50 nm, and g=10 nm. Figure 3b illustrates that resonance occurs at 603 nm, with a transmission rate of 69.53%. The magnetic field distribution at the resonance wavelength of 603 nm is shown in Figure 3c, where the field is predominantly concentrated within Resonator 3. Next, the coupling between Resonator 1 and Resonator 3 is analyzed, focusing on the effect of rotating Resonator 1 on the overall structure.

**Figure 4:**
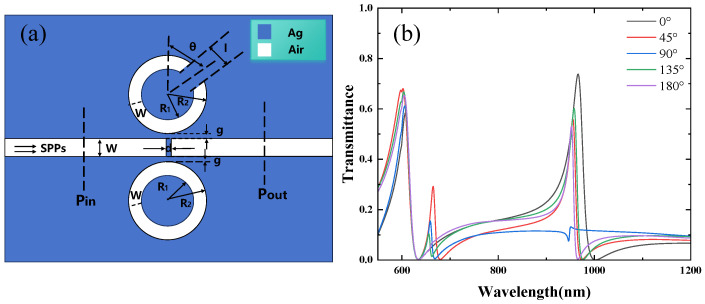
(**a**) Two-dimensional schematic of the MIM waveguide with Resonator 1 and Resonator 3; (**b**) finite element method (FEM) calculations of the transmission spectra of Resonator 1 and Resonator 3.

The structure is shown in Figure 4a, with the parameters $R_{1}=90$ nm, $R_{2}=140$ nm, W=50 nm, g=10 nm, and an initial rotation angle $\theta =45^{{}^{\circ}}$. The rotation angle is incrementally varied in a clockwise direction, from $0^{{}^{\circ}}$ to $45^{{}^{\circ}}$, $90^{{}^{\circ}}$, $135^{{}^{\circ}}$, and $180^{{}^{\circ}}$. Figure 4b displays the transmission spectra for rotation angles ranging from $0^{{}^{\circ}}$ to $180^{{}^{\circ}}$. The simulation results indicate that for each rotation angle, both the first and third Fano resonance peaks appear. Although the positions of these peaks remain unchanged with varying rotation angles, their intensities are observed to vary as the rotation angle changes. The first resonance peak corresponds to Resonator 3, while the third resonance peak is attributed to Resonator 1. As the rotation angle begins to change from $0^{{}^{\circ}}$, the symmetry of the structure is broken, leading to the emergence of a second Fano resonance peak. The position of this peak remains almost unchanged; however, its intensity decreases as the rotation angle increases.

**Figure 5:**
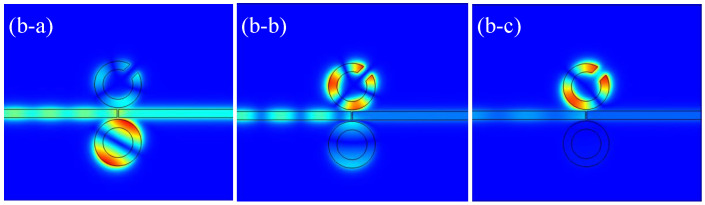
(**b-a**) Magnetic field distribution of the structure at λ=600 nm; (**b-b**) magnetic field distribution of the structure at λ=665 nm; (**b-c**) magnetic field distribution of the structure at λ=955 nm.

Figure 5 shows the magnetic field distribution when Resonator 1 is rotated by $45^{{}^{\circ}}$. At a wavelength of 600 nm, the magnetic field is predominantly concentrated around the ring, confirming that the first resonance peak is associated with Resonator 3. As indicated in [30], the resonance modes of the CSRRC are classified into two types: the symmetric mode at λ=955 nm and the asymmetric mode at λ=665 nm. At 665 nm, the magnetic field is distributed both on the CSRRC and the complete ring, suggesting that the second resonance peak corresponds to the asymmetric mode. At 955 nm, the magnetic field is predominantly concentrated on the CSRRC, indicating that the third resonance peak corresponds to the symmetric mode.

**Figure 6:**
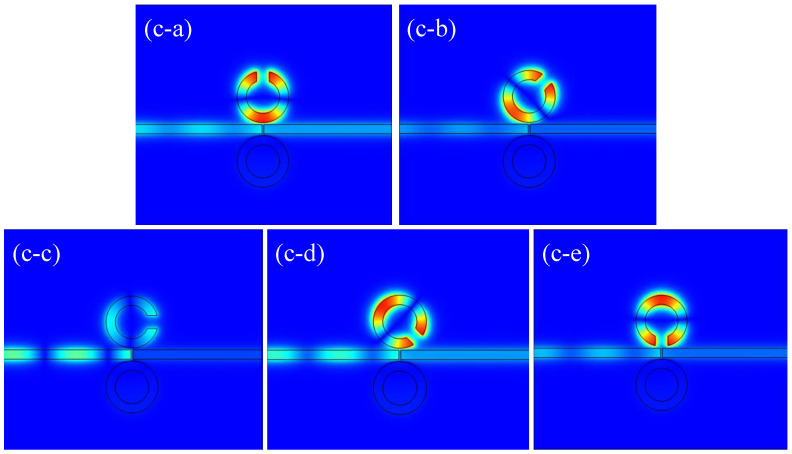
(**c-a**) Magnetic field distribution of the structure at $\theta =0^{{}^{\circ}}$; (**c-b**) magnetic field distribution of the structure at $\theta =45^{{}^{\circ}}$; (**c-c**) magnetic field distribution of the structure at $\theta =90^{{}^{\circ}}$; (**c-d**) magnetic field distribution of the structure at $\theta =135^{{}^{\circ}}$; (**c-e**) magnetic field distribution of the structure at $\theta =180^{{}^{\circ}}$.

**Figure 7:**
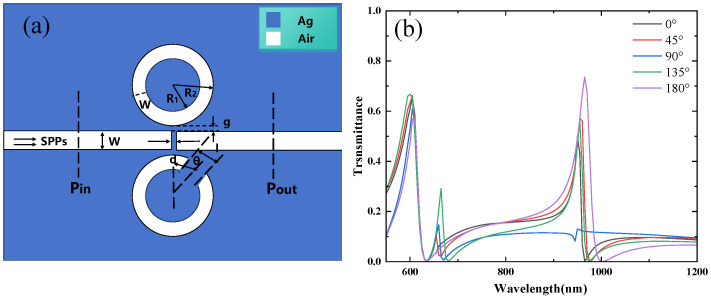
(**a**) Two-dimensional schematic of the MIM waveguide with Resonator 2 and Resonator 3; (**b**) finite element method (FEM) calculation of the transmission spectra of Resonator 2 and Resonator 3.

Additionally, the magnetic field distribution in Figure 6 reveals that the field is minimal when the upper CSRRC is rotated by $90^{{}^{\circ}}$, consistent with the observation in [30], where the transmission rate is lowest at $\theta =90^{{}^{\circ}}$. The magnetic field distributions at other angles are similar, which aligns with the transmission spectra shown in Figure 4b. After analyzing the case where the complete ring is positioned on the lower side of the waveguide, it is also important to consider the scenario where the complete ring is placed on the upper side. The structure is shown in Figure 7a. The parameters of the structure remain unchanged. The rotation angle is varied clockwise from $0^{{}^{\circ}}$ to $45^{{}^{\circ}}$, $90^{{}^{\circ}}$, $135^{{}^{\circ}}$, and $180^{{}^{\circ}}$. Figure 7b shows a curve similar to that in Figure 4b, with the obvious pattern being caused by the symmetry of the structure.

### 3.2. Analysis of the Rotation Directions of the CSRRCs

**Figure 8:**
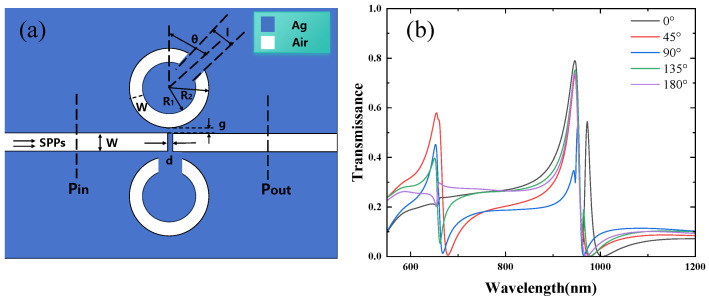
(**a**) Two-dimensional schematic of the MIM waveguide for Resonator 1 and Resonator 2; (**b**) finite element method (FEM) calculation of the transmission spectrum for rotating Resonator 1 and fixing Resonator 2.

In analyzing the simultaneous rotation of Resonators 1 and 2, the effect of rotating the CSRRC on the structure is first considered. The rotation of Resonator 1 is varied while keeping Resonator 2 fixed. The structure is shown in Figure 8a, with the parameters remaining unchanged: $R_{1}=90$ nm, $R_{2}=140$ nm, W=50 nm, g=10 nm, and an initial rotation angle $\theta =45^{{}^{\circ}}$. The rotation angle is incrementally varied in a clockwise direction from $0^{{}^{\circ}}$ to $45^{{}^{\circ}}$, $90^{{}^{\circ}}$, $135^{{}^{\circ}}$, and $180^{{}^{\circ}}$. Figure 8b displays the transmission spectra for Resonator 1 rotated from $0^{{}^{\circ}}$ to $180^{{}^{\circ}}$. Comparing it with Figure 4b, the first resonance peak at 603 nm, which corresponds to Resonator 3, disappears.

**Figure 9:**
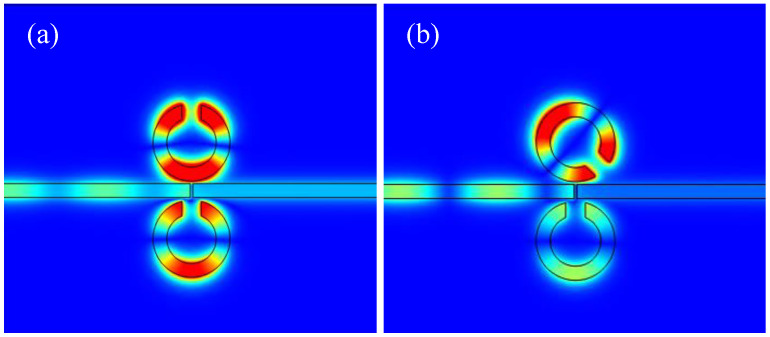
(**a**) Magnetic field distribution of the structure at $\theta =0^{{}^{\circ}}$; (**b**) magnetic field distribution of the structure at $\theta =135^{{}^{\circ}}$.

After the coupling shifts from Resonator 3 to Resonator 2 on the lower side of the waveguide, the transmission rate of the second resonance peak increases. This is because the second peak arises from the asymmetric mode of the CSRRC. The third resonance peak in Figure 8b is largely consistent with the one in Figure 4b, with the minimum transmission occurring at $\theta =90^{{}^{\circ}}$ and the transmission remaining nearly unchanged for the other rotation angles, in agreement with the magnetic field distribution in Figure 9a,b. A fourth resonance peak appears at $\theta =0^{{}^{\circ}}$ and $135^{{}^{\circ}}$.

**Figure 10:**
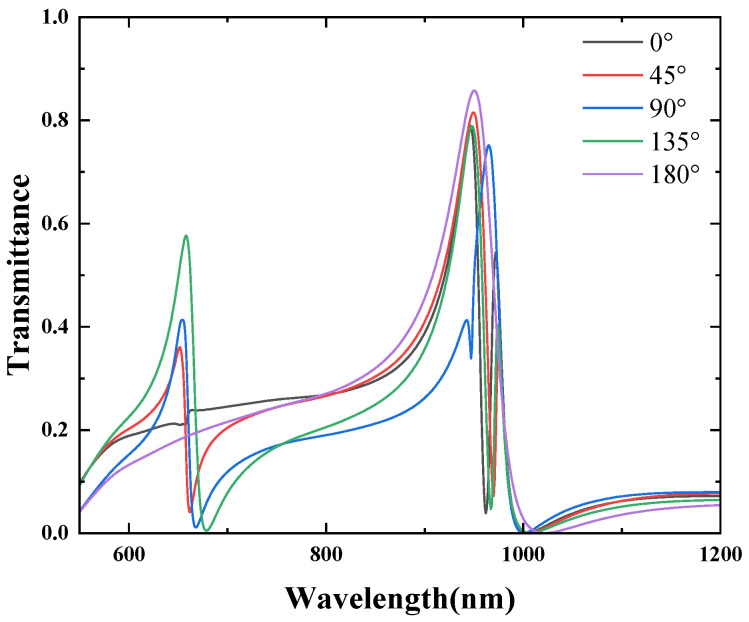
Finite element method (FEM) calculation of the transmission spectrum for a fixed Resonator 1 and a rotating Resonator 2.

Since the structure is symmetric with respect to the x-axis, when analyzing the effect of a single rotation angle on the structure, Resonator 1 is kept fixed while the rotation angle of Resonator 2 is varied. The parameters remain unchanged: $R_{1}=90$ nm, $R_{2}=140$ nm, W=50 nm, g=10 nm, and an initial rotation angle $\theta =45^{{}^{\circ}}$. The rotation angle is incrementally varied clockwise from $0^{{}^{\circ}}$ to $45^{{}^{\circ}}$, $90^{{}^{\circ}}$, $135^{{}^{\circ}}$, and $180^{{}^{\circ}}$. Figure 10 shows the transmission spectra for Resonator 2 rotated from $0^{{}^{\circ}}$ to $180^{{}^{\circ}}$. When compared with the transmission spectra in Figure 8b, the overall trend of the curves remains nearly unchanged. Notably, a fourth resonance peak appears at $\theta =45^{{}^{\circ}}$ and $\theta =135^{{}^{\circ}}$. At $\theta =180^{{}^{\circ}}$, the second resonance peak disappears. This disappearance is attributed to the x-axis symmetry of the CSRRCs, which results in the vanishing of the second resonance peak that arises from the asymmetric mode.

**Figure 11:**
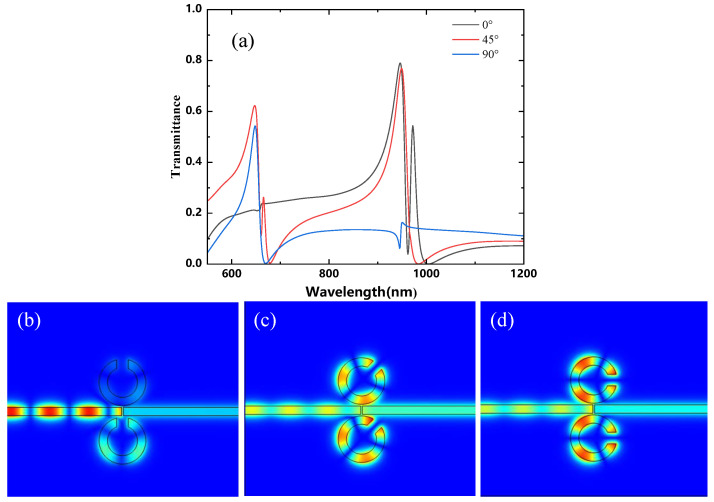
(**a**) Finite element method (FEM) calculation of simultaneous rotational transmission spectra for Resonators 1 and 2; (**b**) magnetic field distribution of the structure at $\theta =0^{{}^{\circ}}$; (**c**) magnetic field distribution of the structure at $\theta =45^{{}^{\circ}}$; (**d**) magnetic field distribution of the structure at $\theta =90^{{}^{\circ}}$.

The effects of simultaneously rotating both Resonators 1 and 2 on the structure are discussed below. Since the structure is symmetric with respect to the x-axis, it is sufficient to consider rotation angles of $\theta =0^{{}^{\circ}}$, $45^{{}^{\circ}}$, and $90^{{}^{\circ}}$. The structure is depicted in Figure 11a, with the following unchanged parameters: $R_{1}=90$ nm, $R_{2}=140$ nm, W=50 nm, g=10 nm, and $\theta =45^{{}^{\circ}}$. The rotation angle θ is varied in a clockwise direction, with values of $0^{{}^{\circ}}$, $45^{{}^{\circ}}$, and $90^{{}^{\circ}}$. Figure 11a shows the transmission spectra for the simultaneous rotation of both Resonators 1 and 2 and Figure 11b–d show the magnetic field distribution of the structure at $\theta =0^{{}^{\circ}}$, $\theta =45^{{}^{\circ}}$, and $\theta =90^{{}^{\circ}}$. The first resonance peak increases in intensity due to changes in asymmetry, and a new second resonance peak appears at the center when $\theta =45^{{}^{\circ}}$. When $\theta =45^{{}^{\circ}}$, the transmission rate of the structure is higher, making it easier to observe. Therefore, this study focuses on analyzing the structure with an orientation angle of $\theta =45^{{}^{\circ}}$.

**Figure 12:**
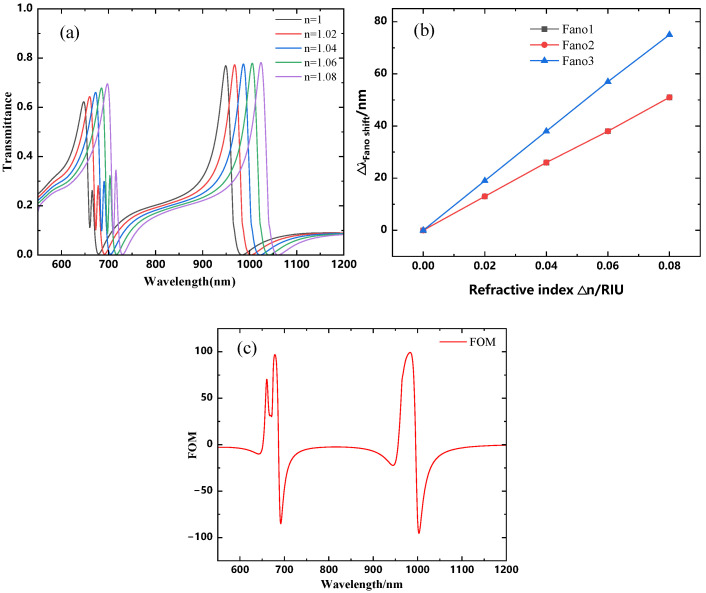
(**a**) Transmission spectra of the sensor at different refractive indices n; (**b**) fitted line graph of peak shift (∆λ) varying with the refractive index (∆n); (**c**) FOM values corresponding to each resonance peak.

The above study shows that the Fano resonance is excited throughout the structure at the resonance wavelength. Also, the asymmetric line shape of the Fano resonance makes its resonance wavelength extremely sensitive to refractive index changes [33]. The extremely narrow transmission peaks produced by the Fano resonance can markedly enhance the sensing resolution. As a result, a more in-depth analysis of the impact of the medium’s refractive index on this structure is warranted. Figure 12a displays the transmission spectra of the structure at different refractive indices, where the refractive index varies from 1.00 to 1.08 RIU (with a step size of 0.02), while the other waveguide parameters remain unchanged. As shown in this figure, with an increase in the refractive index, all three Fano resonances exhibit a noticeable redshift. As the change in refractive index (∆n) increases, the resonance wavelengths shift, as illustrated in the figure. The figure of merit (FOM) [34] and sensitivity (S) [35] are two key parameters for evaluating the sensor performance. These are generally defined as follows:

(2) $$S=\frac{∆\lambda }{∆n}$$

(3) $$FOM=\frac{∆T/∆n}{T}$$

wherein $T=\left(T_{1.00}+T_{1.02}\right)/2$, $∆T=T_{1.02}-T_{1.00}$.

As observed in Figure 12a, the shift in the resonance peaks shows a linear variation with the increase in the refractive index, which suggests potential applications for refractive index sensors. Figure 12b,c present the results of linear fitting, revealing that the sensitivity of the first Fano resonance peak is 642.71 nm/RIU, with an FOM of 69.84. The sensitivity of the second Fano resonance peak is also 642.71 nm/RIU, but with a higher FOM of 96.97. The sensitivity of the third Fano resonance peak is 946.88 nm/RIU, with an FOM of 99.17.

## 4. Analysing the CSRRCs of Different Structural Dimensions

**Figure 13:**
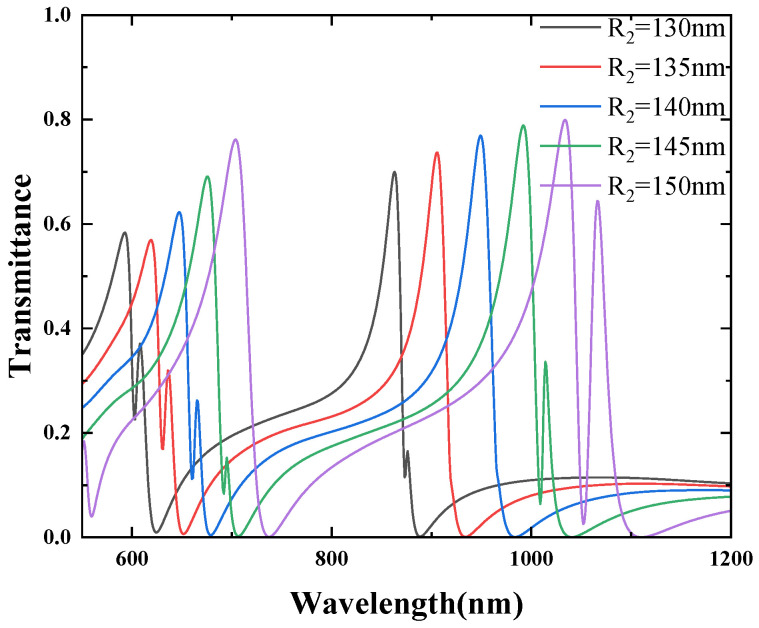
Effect of different $R_{2}$ on structural Fano resonance.

In general, the transmission characteristics of MIM waveguide structures are influenced by changes in their structural parameters. In this study, Fano resonance is generated due to the coupling between the MIM stub and the double CSRRCs. As a result, the peak intensity and line shape of the Fano resonance are affected by the structural geometric parameters. We further investigated the impact of geometric parameters on Fano resonance. The values of these geometrical parameters are the result of optimization, choosing the geometrical parameters associated with the highest transmittance and also with reference to previous research in [30]. Firstly, the other parameters of the waveguide structure were fixed as d=10 nm, l=70 nm, g=10 nm, and W=50 nm, while the outer radius $R_{2}$ of the CSRRC was increased from 130 nm to 150 nm in increments of 5 nm. The influence of this single variable on the Fano resonance was then studied. The transmission spectra of the structure for different values of the outer radius are shown in Figure 13. As $R_{2}$ increases, all three Fano resonance peaks exhibit a noticeable redshift, indicating that the resonance wavelength of the Fano peaks is determined by the narrow-band discrete states. The increase in $R_{2}$ results in a shift in the resonance wavelengths in the narrow-band spectrum, which ultimately causes the redshift of the Fano resonance.

**Figure 14:**
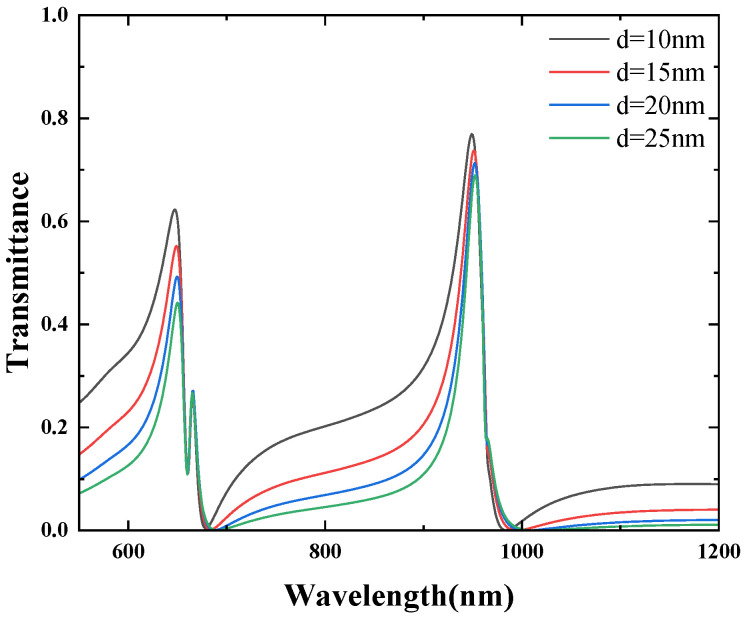
Effect of different d on Fano resonance.

Additionally, the effect of the silver stub width on the transmission spectra was investigated. The stub width d was varied between 10 nm and 25 nm in increments of 5 nm, while keeping the other parameters unchanged. The corresponding transmission spectra are shown in Figure 14. As observed in this figure, the positions of the Fano resonance peaks remain nearly unchanged. However, the first and third Fano resonance peaks gradually decrease in intensity with increasing d, while the second Fano resonance peak remains unaffected. This behavior can be attributed to the fact that the resonance wavelength is primarily determined by the geometric parameters of the CSRRC. As the stub width d increases, the line shape of the spectrum also changes. This is due to the fact that Fano resonance arises from the interaction between a broad-band continuum state and a narrow-band discrete state. As the stub width increases, the wide continuous spectrum generated by the stub in the MIM waveguide changes, which in turn affects the shape of the Fano resonance curve. For the effect of d and R, it that seems the transmission rate of each Fano resonance peak slightly changes. The radius $R_{2}$ increases from 130 nm to 150 nm in increments of 5 nm, and each $R_{2}$ corresponds to an FOM of 98.5, 98.70, 99.17, 99.54, and 99.10, respectively. The d increases from 10 nm to 25 nm in increments of 5 nm, and each d corresponds to an FOM of 99.17, 99.06, 98.8, and 99.04, respectively, The effect of these parameters on FOM is small and almost unchanged.

**Figure 15:**
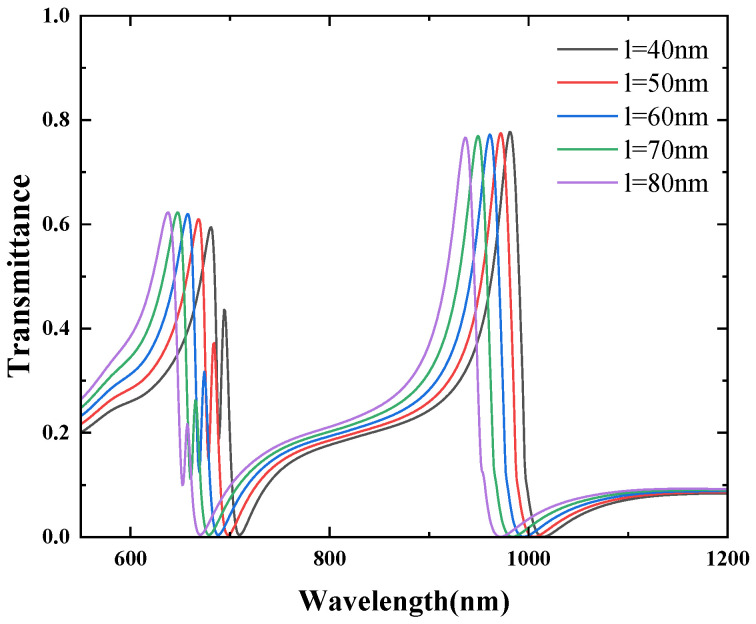
Effect of different l on Fano resonance.

**Figure 16:**
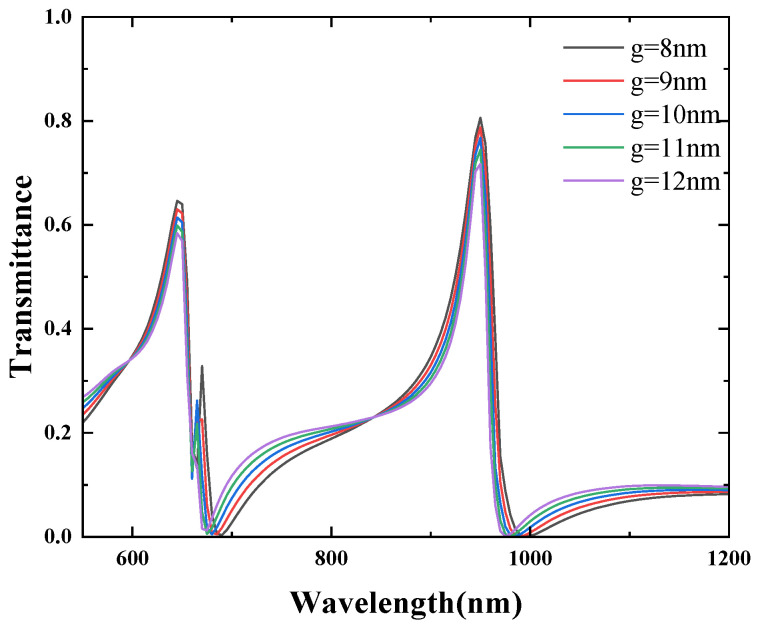
Effect of different g on Fano resonance.

Finally, the effect of the CSRRC gap length l on the transmission spectra was studied. The other structural parameters were kept constant, and the gap length l was varied from 40 nm to 80 nm in increments of 10 nm. The simulation results of the structure are shown in Figure 15. As l increases, a noticeable blueshift of the Fano resonance peaks is observed. However, the transmission rate of each Fano resonance peak remains almost unchanged. The effect of the direct distance between the CSRRC and the waveguide was studied. The other structural parameters were kept constant, and the g was varied from 8 nm to 12 nm in increments of 1 nm. The simulation results of the structure are shown in Figure 16. As g increases, the position of each the Fano resonance peaks remain almost unchanged, but the transmittance decreases sequentially, but by a small amount.

## 5. Conclusions

In this work, a structure with high-quality performance was designed and numerically investigated using the FEM method. The structural performance is presented through the values of sensitivity and FOM. The highest transmission rate achieved in this study is 76.73%, with a sensitivity of 946.88 nm/RIU and an FOM = 99.17. The numerical results demonstrate that varying the rotation angles of the CSRRCs on both sides of the MIM waveguide can produce different resonance wavelengths within the electromagnetic spectrum range of 550 nm to 1200 nm. By altering the coupling between different resonator structures, along with changing the rotation angles and geometric parameters, various Fano resonance transmission peaks can be obtained. This study primarily investigates the effects of coupling different resonators and variations in rotation angle parameters on the performance of MIM-based Fano resonance optical sensors. The results indicate the following: (1) coupling different resonators significantly impacts the Fano resonance transmission peak, leading to changes in transmission efficiency; (2) altering the rotation angle of the structure changes the transmission efficiency of the Fano resonance peak, while the position of the transmission peak remains nearly unchanged; and (3) modifying the geometric parameters of the structure allows for the tuning of the Fano resonance wavelength. These findings are of considerable importance for subsequent research and practical applications.

## Data Availability

The original contributions presented in this study are included in the article. Further inguiries can be directed to the corresponding authors.
